# Supplementary figures and images for: A modular platform to display multiple hemagglutinin subtypes on a single immunogen
Source: eLife. 2025 Dec 8;13:RP97364. doi: 10.7554/eLife.97364 (PMC12685301; doi:10.7554/eLife.97364)

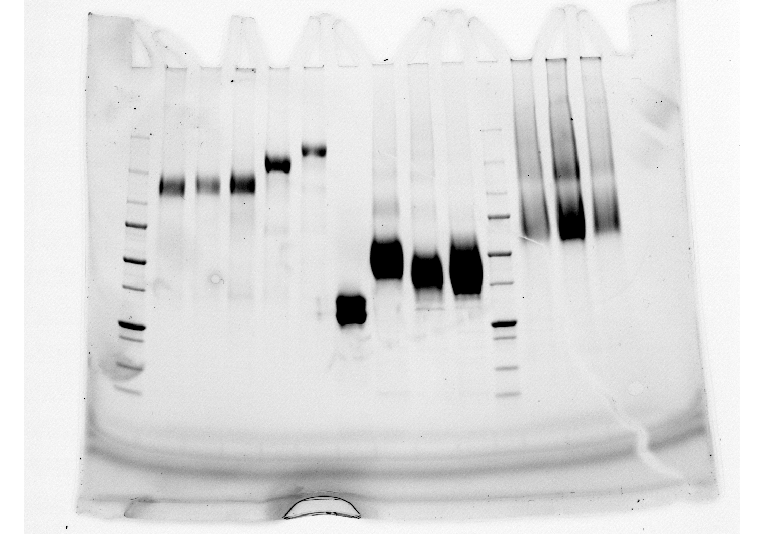

Supplement: Figure 1—figure supplement 1—source data 1. [file elife-97364-fig1-figsupp1-data1.zip › Figure 1- figure supplement 1 - source data 1.tif]

WT H3/B/H1 3-mer BoaS

2XGSS  
3XGSS  
4XGSS

250kD-

150kD-

100kD-

75kD-

50kD-

37kD-

25kD-

20kD-

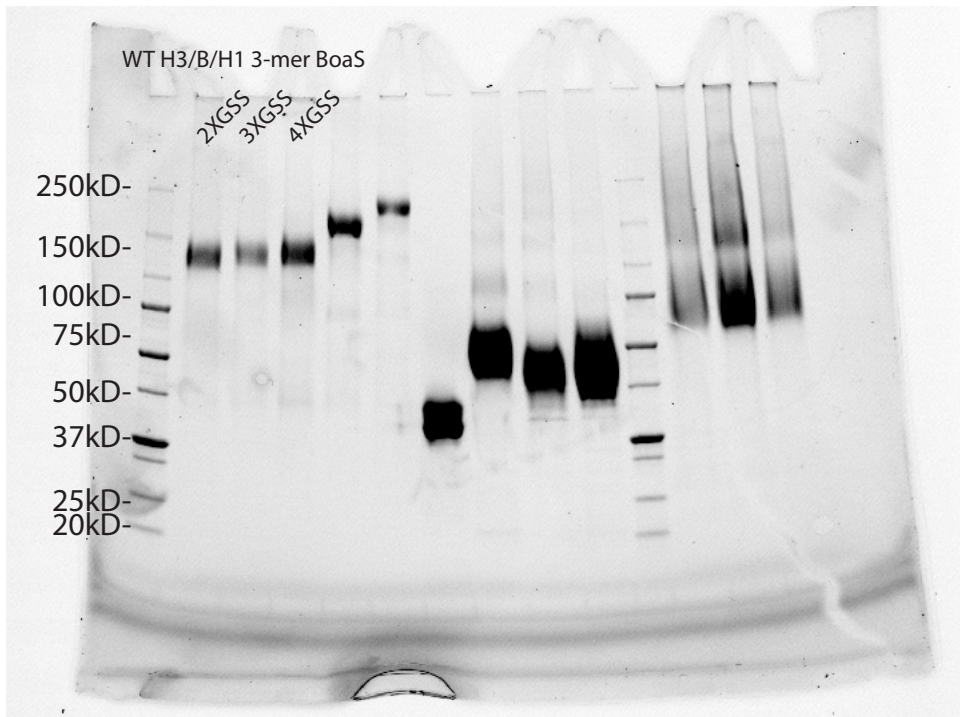

Supplement: Figure 1—figure supplement 1—source data 2. [file elife-97364-fig1-figsupp1-data2.zip › Figure 1- figure supplement 1 - source data 2.pdf]

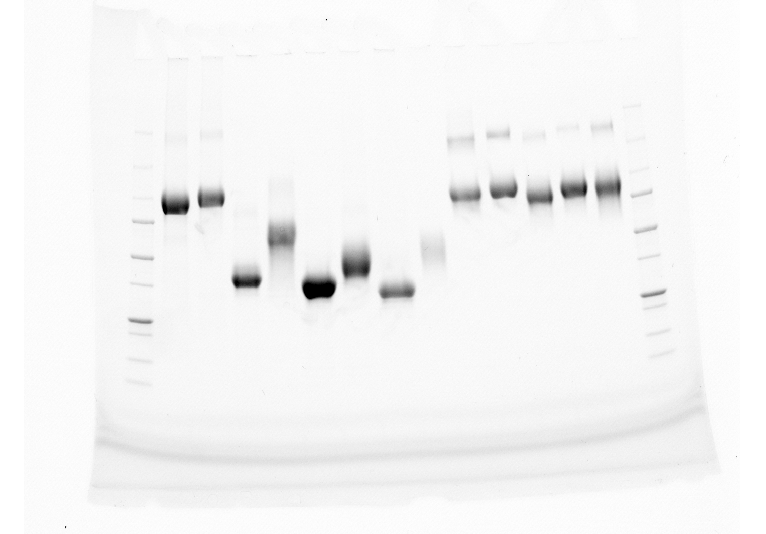

Supplement: Figure 1—figure supplement 2—source data 1. [file elife-97364-fig1-figsupp2-data1.zip › Figure 1- figure supplement 2- source data 1.tif]

WT H9/H5/H7 BoaS  
WT H5/H9/H7 BoaS

250kD-

150kD-

100kD-

75kD-

50kD-

37kD-

25kD-

20kD-

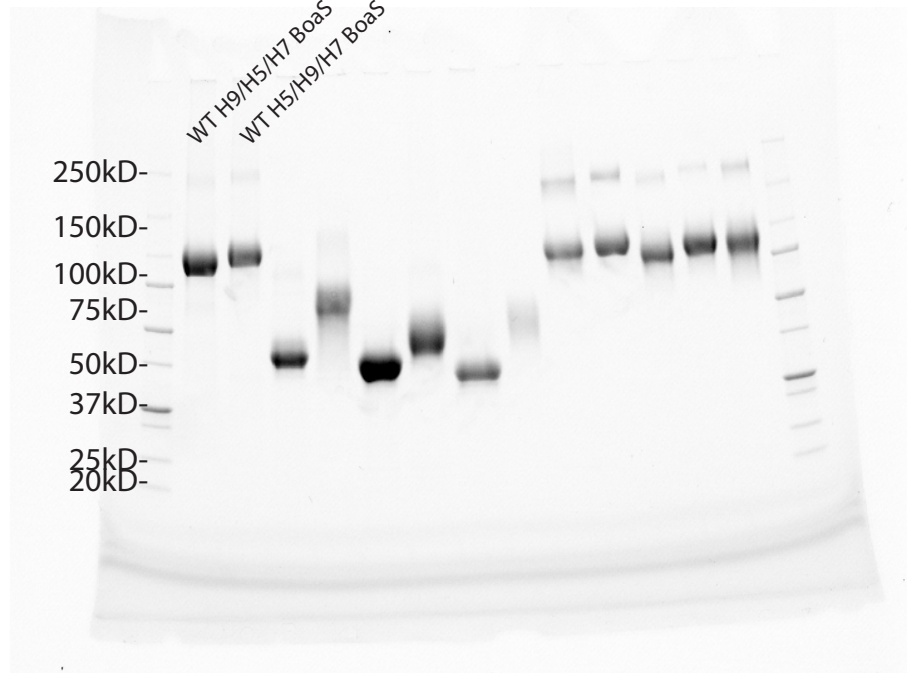

Supplement: Figure 1—figure supplement 2—source data 2. [file elife-97364-fig1-figsupp2-data2.zip › Figure 1- figure supplement 2- source data 2.pdf]

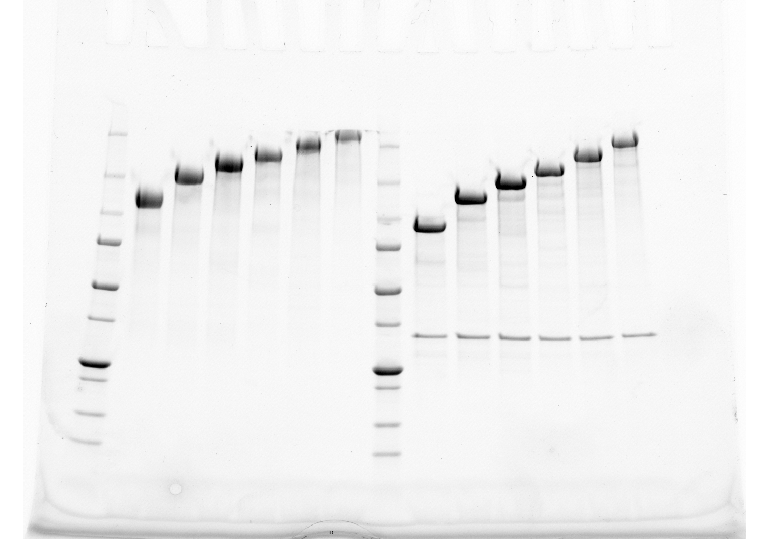

Supplement: Figure 2—source data 1. [file elife-97364-fig2-data1.zip › Figure 2 - source data 1.tif]

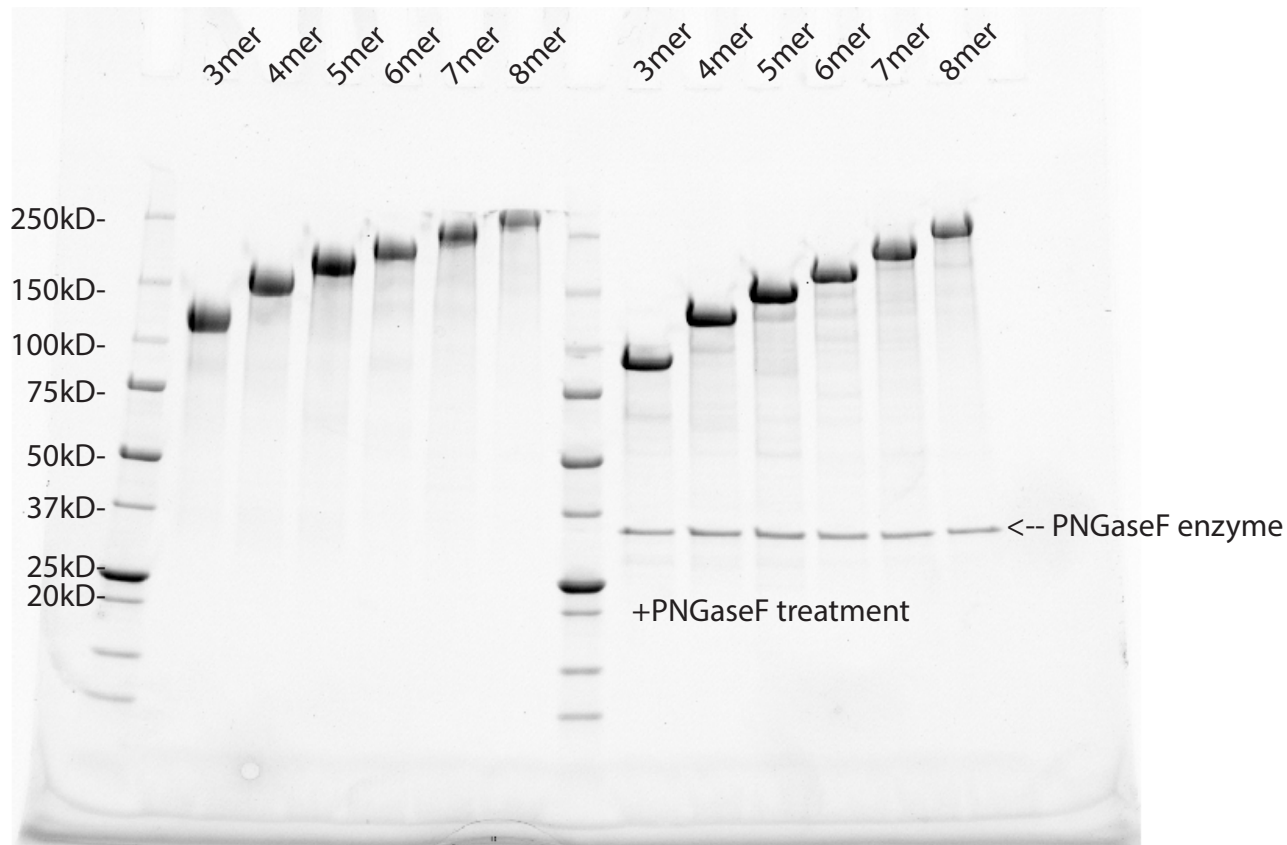

Supplement: Figure 2—source data 2. [file elife-97364-fig2-data2.zip › Figure 2 - source data 2.pdf]

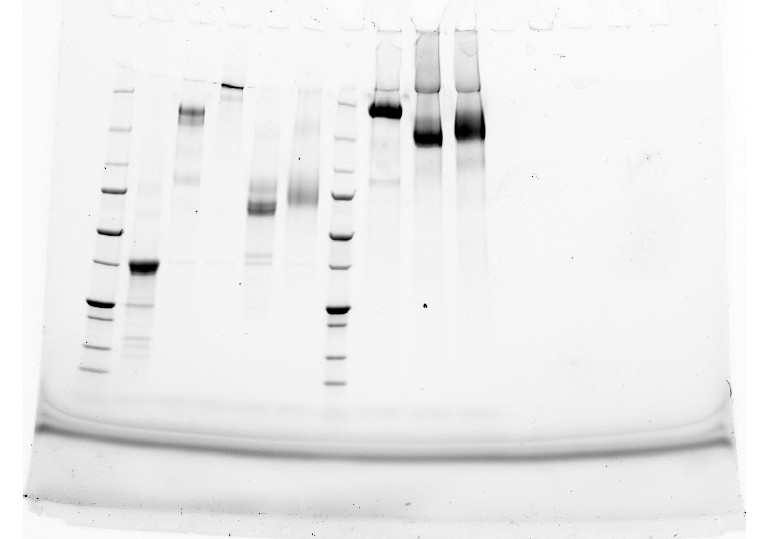

Supplement: Figure 5—figure supplement 1—source data 1. [file elife-97364-fig5-figsupp1-data1.zip › Figure 5- figure supplement 1 - source data 2.tif]

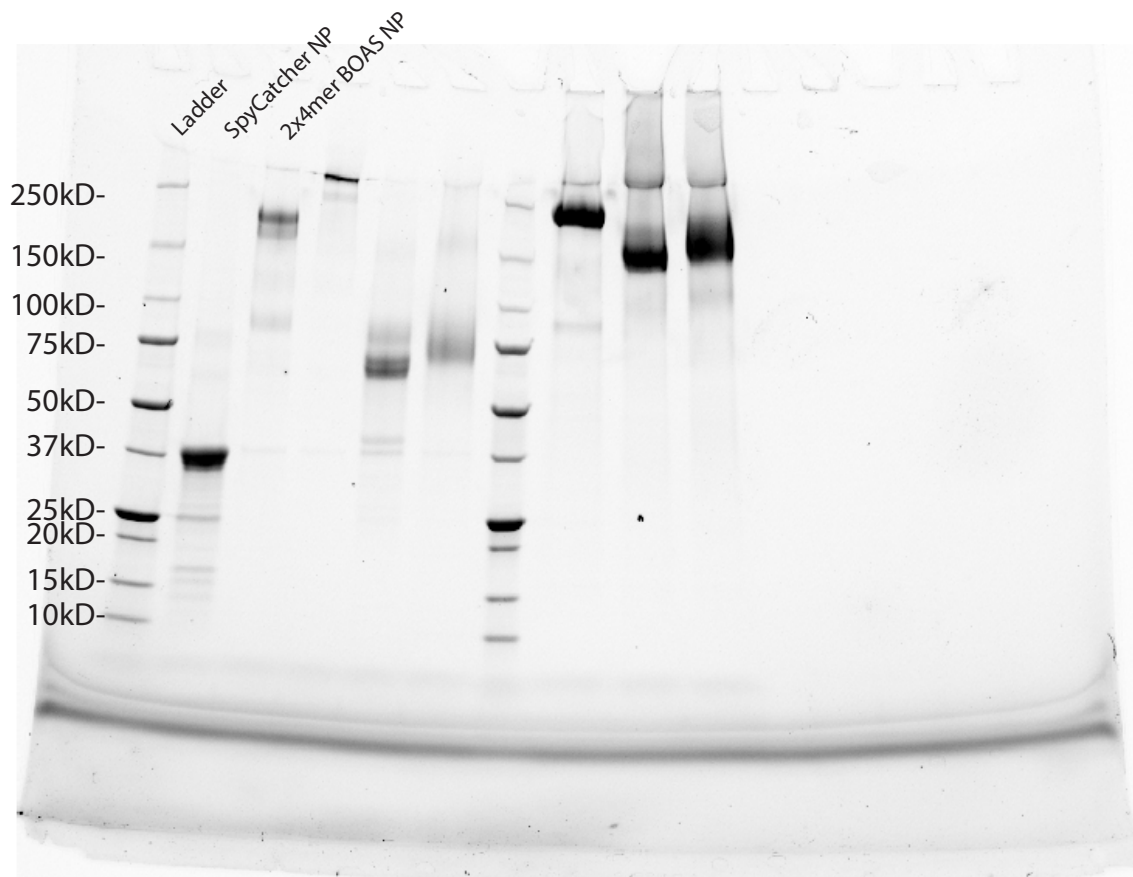

Supplement: Figure 5—figure supplement 1—source data 2. [file elife-97364-fig5-figsupp1-data2.zip › Figure 5- figure supplement 1 - source data 1.pdf]
