# Supplementary material for: A modular platform to display multiple hemagglutinin subtypes on a single immunogen
Source: eLife. 2025 Dec 8;13:RP97364. doi: 10.7554/eLife.97364 (PMC12685301; doi:10.7554/eLife.97364)
Supplement: Figure 2—source data 3. [file elife-97364-fig2-data3.pdf]

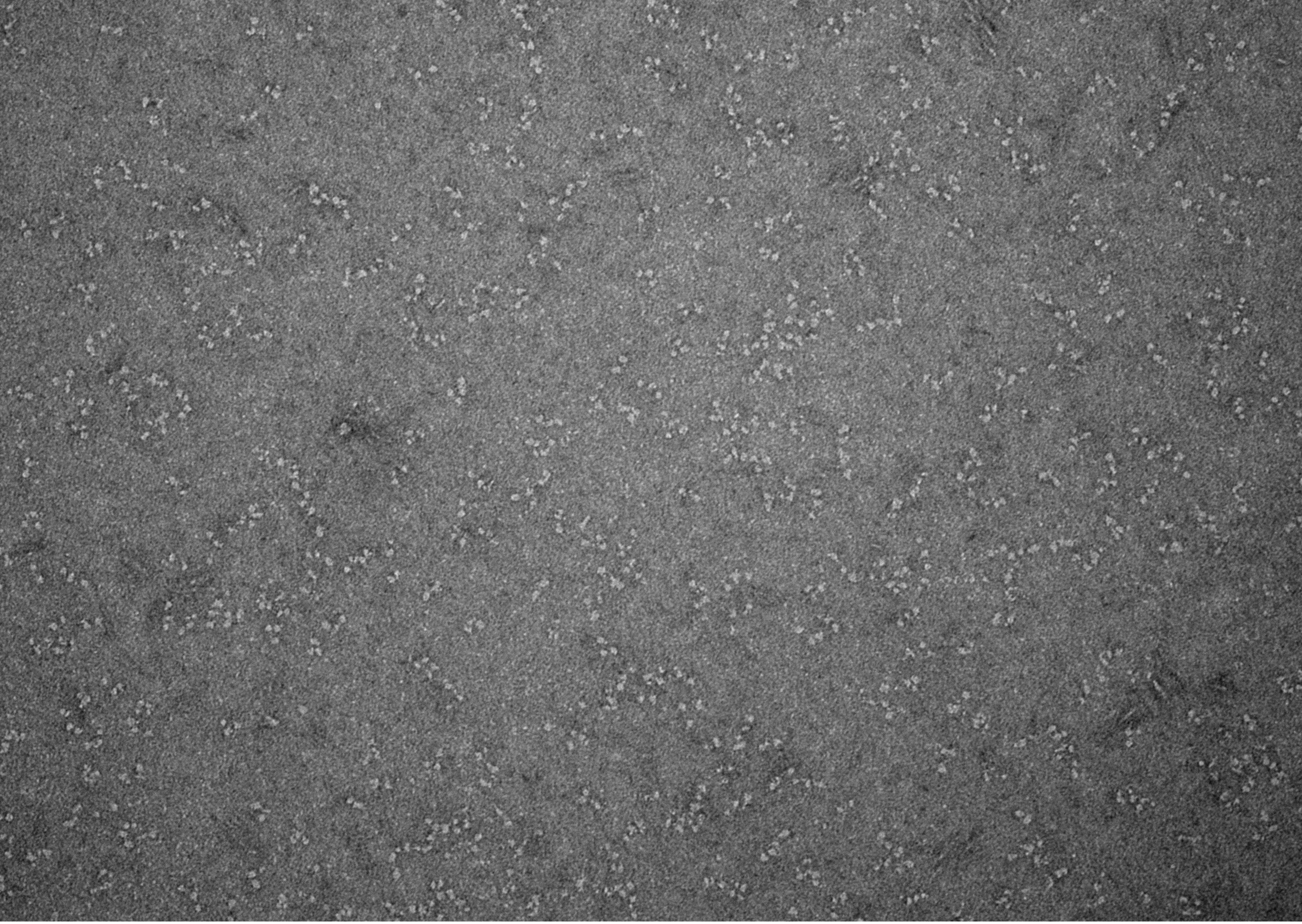

Dana_Ragon_081722_007.tif
3mer_4XGSSS_H3HBH1_BoaS

11:02 2022-08-17
TEM Mode: Imaging

Camera: NANOSPRT43, Exposure: 600 (ms) x 3 drift frames, Gain: 10, Bin: 1
Gamma: 1.00, No Sharpening, Normal Contrast

100 nm
HV=80kV
Direct Mag: 49000 x
X:206.1725822 Y: -193.0188744
Tilt:0.00
AMT Camera System
