# Supplementary material for: A modular platform to display multiple hemagglutinin subtypes on a single immunogen
Source: eLife. 2025 Dec 8;13:RP97364. doi: 10.7554/eLife.97364 (PMC12685301; doi:10.7554/eLife.97364)
Supplement: Figure 5—source data 1. [file elife-97364-fig5-data1.zip › Figure 5D source data- Negative stain EM 2.pdf]

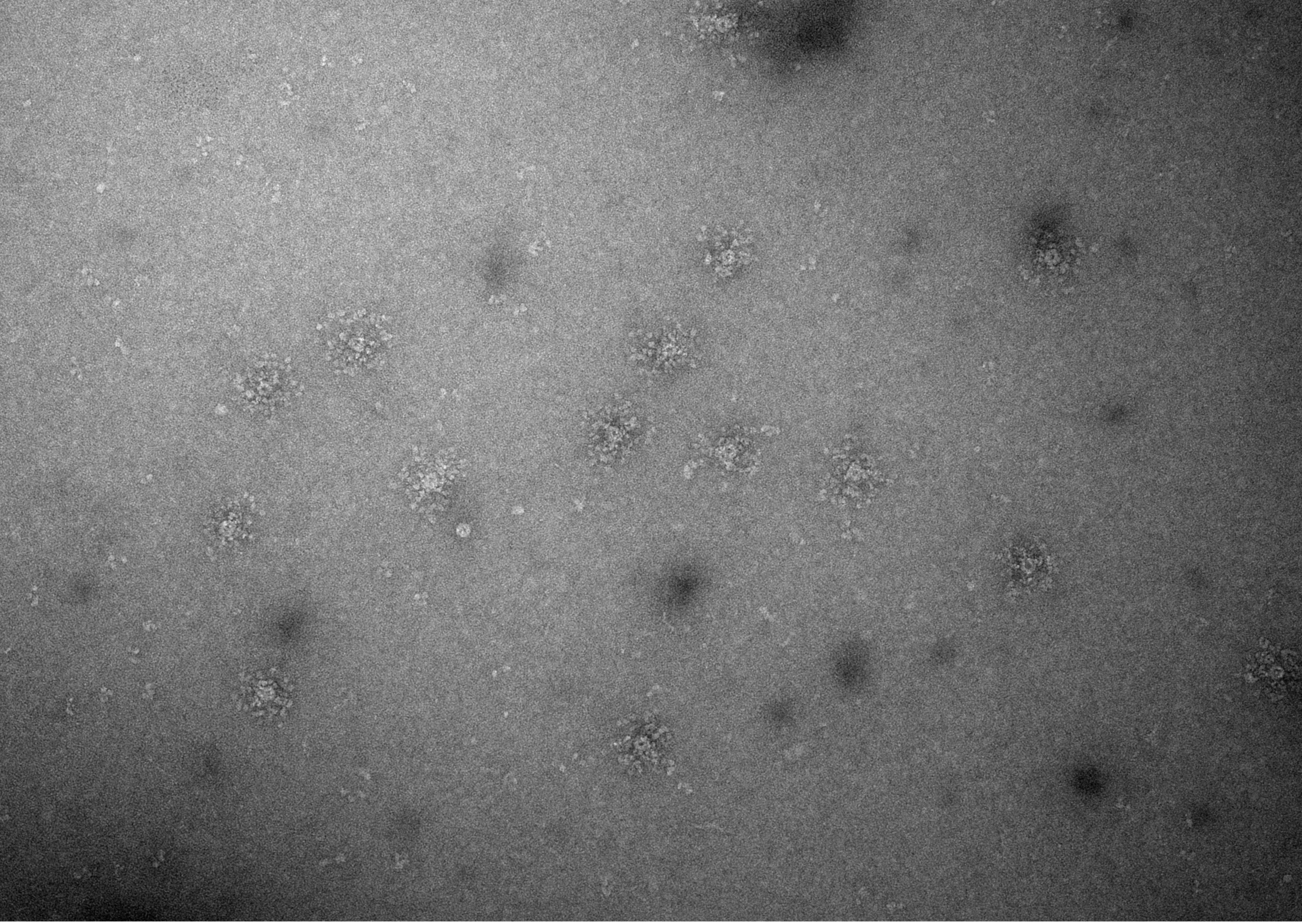

Dana Thornlow_060723_#4_003.tif
4. WT 2x4mer NP_20ug/ml

Cal: 0.000143 µm/pix
15:56 2023-06-07
TEM Mode: Imaging
Camera: NANOSPRT43, Exposure: 600 (ms) x 3 std. frames, Gain: 10, Bin: 1
Gamma: 1.00, No Sharpening, Normal Contrast

100 nm
HV=80kV
Direct Mag: 49000 x
AMT Camera System
