# Supplementary material for: A modular platform to display multiple hemagglutinin subtypes on a single immunogen
Source: eLife. 2025 Dec 8;13:RP97364. doi: 10.7554/eLife.97364 (PMC12685301; doi:10.7554/eLife.97364)
Supplement: Figure 5—source data 1. [file elife-97364-fig5-data1.zip › Figure 5D source data- Negative Stain EM copy.pdf]

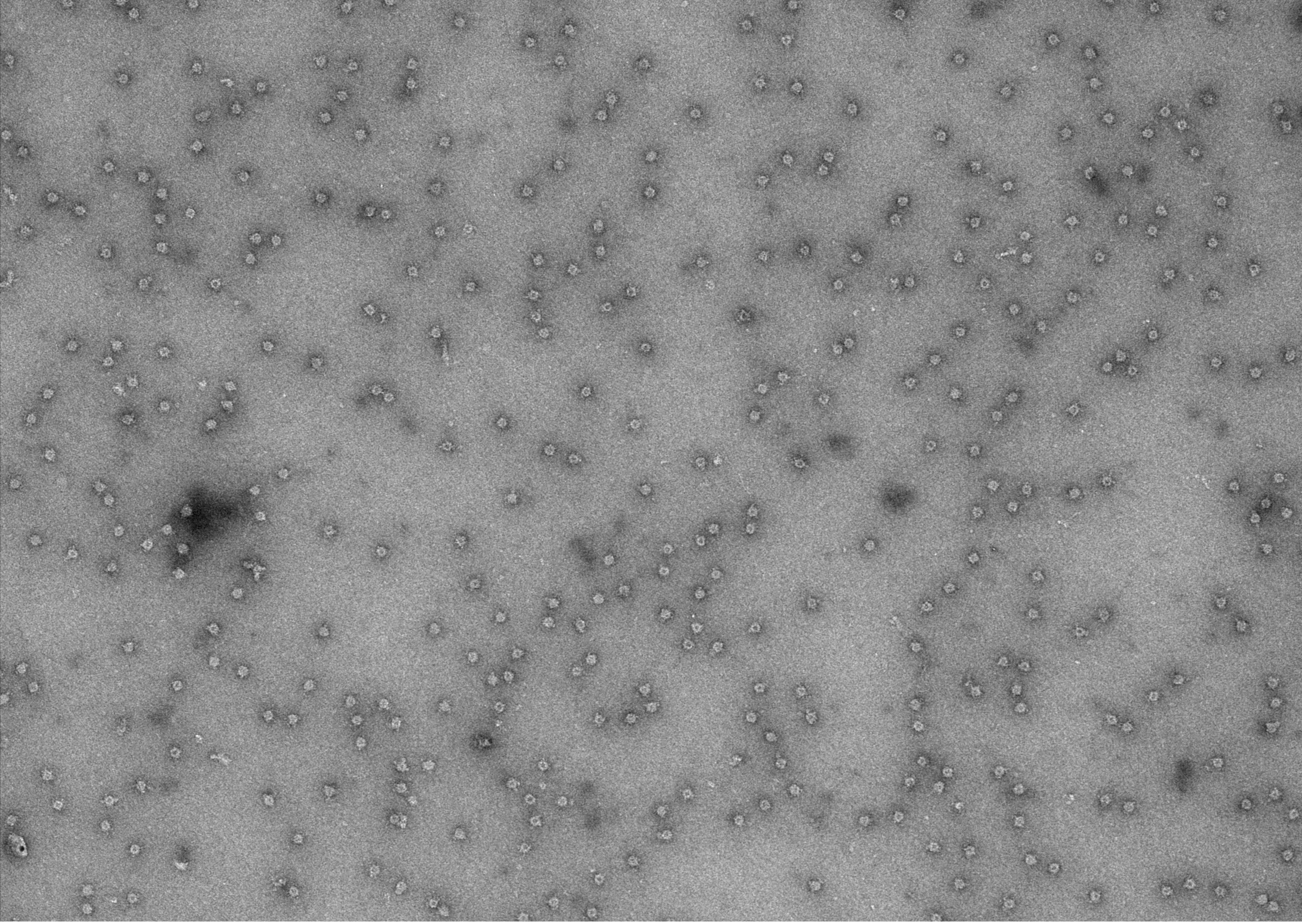

Dana Thornlow_060723_#1_004.tif
#1 SpyCatcher NP_20 ug/ml
0.75% UF

Cal: 0.000234 µm/pix
14:58 2023-06-07
TEM Mode: Imaging
Camera: NANOSPRT43, Exposure: 600 (ms) x 3 drift frames, Gain: 10, Bin: 1
Gamma: 1.00, No Sharpening, Normal Contrast

200 nm
HV=80kV
Direct Mag: 30000 x
AMT Camera System
